# Neutropenia and Lymphopenia in Systemic Lupus Erythematosus: Distinct Phenotypes and Associated Factors in a Saudi Multicenter Study

**DOI:** 10.3390/jcm15166481

**Published:** 2026-08-21

**Authors:** Roaa Aljohani, Ghada Aljanobi, Khawla K. Alghanim, Nadeem Butt, Alaa Ahmed, Farah Alabbasi, Samar Alharbi

**Affiliations:** 1 Department of Internal Medicine, College of Medicine, Taibah University, Madinah 41411, Saudi Arabia; skhattabi@taibahu.edu.sa; 2 Rheumatology Unit, Johns Hopkins Aramco Healthcare, Dhahran 34465, Saudi Arabia; dr.ghadaaljanobi@gmail.com (G.A.); farah.mohamed@jhah.com (F.A.); 3 Rheumatology Unit, Department of Medicine, King Fahd Military Medical Complex, Dhahran 31932, Saudi Arabia; dr.khawla25@yahoo.com; 4 Department of Family and Community Medicine, King Abdulaziz University Rabigh, Jeddah 25732, Saudi Arabia; nshafique@kau.edu.sa; 5 Rheumatology Unit, King Salman Bin Abdulaziz Medical City, Medina 42319, Saudi Arabia; dr.alaa.alsawi@gmail.com

**Keywords:** leukopenia, lymphopenia, neutropenia, systemic lupus erythematosus, Saudi Arabia

## Abstract

**Background/Objectives**: Neutropenia and lymphopenia are common hematologic manifestations of systemic lupus erythematosus (SLE), but whether they represent distinct phenotypes is not well established. This study evaluated their prevalence, patterns, and associated factors. **Methods**: This multicenter retrospective study included 353 adults with SLE from three Saudi centers. Cytopenias were defined using prespecified laboratory criteria. Patients were classified as having neither abnormality, isolated neutropenia, isolated lymphopenia, or both. Multivariable logistic regression was used to assess factors associated with ever-neutropenia and ever-lymphopenia. **Results**: Leukopenia occurred in 141 patients (39.9%). Ever-neutropenia and ever-lymphopenia were each observed in 88 patients (24.9%); 47 (13.3%) had isolated neutropenia, 47 (13.3%) had isolated lymphopenia, and 41 (11.6%) had both abnormalities. Persistent neutropenia and lymphopenia occurred in 22 (6.2%) and 23 (6.5%) patients, respectively. Most neutropenia was mild, and only three patients had severe neutropenia. The four phenotypes differed in age, body mass index, autoimmune hemolytic anemia, platelet count, anti-Smith positivity, and immunosuppressive exposure. No included variable was independently associated with ever-neutropenia. Ever-lymphopenia was independently associated with male sex (aOR 2.66, 95% CI 1.22–5.81), anti-Smith positivity (aOR 2.71, 95% CI 1.40–5.24), and exposure to azathioprine (aOR 2.07, 95% CI 1.16–3.71), mycophenolate mofetil use (aOR 2.79, 95% CI 1.42–5.52), and rituximab use (aOR 3.83, 95% CI 1.50–9.75). **Conclusions**: Neutropenia and lymphopenia each occurred in one-quarter of patients with SLE, whereas persistent cytopenias were uncommon. No independent associations were identified for ever-neutropenia. The demographic, serologic, and treatment-related associations of ever-lymphopenia support separate evaluation of the two abnormalities in SLE.

## 1. Introduction

Systemic lupus erythematosus (SLE) is a chronic multisystem autoimmune disease characterized by variable clinical presentations and diverse immunologic, hematologic, and organ-specific manifestations that have a significant impact on patients’ quality of life [1,2]. Hematologic abnormalities are among the most common features of SLE and are recognized as part of the disease phenotype in clinical practice and established classification criteria [3,4,5].

Leukopenia, lymphopenia, and neutropenia may occur in SLE as a result of active disease, immune-mediated peripheral cell destruction, bone marrow suppression, or medication-related toxicity [6,7,8,9,10,11,12]. A systematic review by Carli et al. reported leukopenia in 22–41.8% of patients with SLE, lymphopenia in 15–82%, and neutropenia in 20–40% [13]. A systematic review of observational studies in Saudi Arabia also reported marked variability in the frequency of leukopenia, which ranged from 15.6% to 58.7%, likely because of differences in definitions, patient populations, and disease severity [14].

A large prospective cohort study showed that although lymphopenia and neutropenia are common in SLE, they differ in their associations with disease activity and medication exposure, thus supporting the need to evaluate them as distinct entities rather than grouping them under the broad category of leukopenia [15]. Lymphopenia has been more consistently associated with clinical and serologic disease activity. However, corticosteroids and immunosuppressive therapies may also contribute [15,16,17,18,19]. Neutropenia in SLE may similarly arise from immune-mediated mechanisms, including anti-neutrophil antibodies, anti-Ro-associated neutrophil binding, and complement activation, as well as from treatment-related bone marrow suppression [9,15,20,21]. Previous cohort studies further indicate that neutropenia has clinical and treatment-related associations distinct from those of lymphopenia [15,21].

Most studies have examined leukopenia as a single hematologic manifestation, and relatively few have evaluated isolated neutropenia, isolated lymphopenia, combined phenotypes, or persistent abnormalities separately. Data from Saudi Arabia are particularly limited regarding the prevalence and associated factors of neutropenia and lymphopenia in SLE. Therefore, this multicenter study aimed to determine the prevalence and patterns of neutropenia and lymphopenia and to identify factors independently associated with ever-neutropenia and ever-lymphopenia.

## 2. Materials and Methods

### 2.1. Study Design and Setting

This multicenter retrospective cohort study included adult patients with SLE who were followed at three centers in Saudi Arabia (King Salman Bin Abdulaziz Medical City, Medina, Saudi Arabia; Johns Hopkins Aramco Healthcare, Dhahran, Saudi Arabia; and King Fahd Military Medical Complex, Dhahran, Saudi Arabia). The study was reported in accordance with the Strengthening the Reporting of Observational Studies in Epidemiology (STROBE) statement for cohort studies [22].

### 2.2. Study Population and Data Collection

Patients were eligible for inclusion if they were aged 18 years or older and fulfilled either the 1997 American College of Rheumatology classification criteria or the 2012 Systemic Lupus International Collaborating Clinics classification criteria [3,4]. Patients were excluded if they had hematologic malignancy, bone marrow failure, or a primary hematologic disorder unrelated to SLE.

Adult patients with SLE who were followed at the participating centers between January 2018 and February 2026 were screened for eligibility. Data were extracted retrospectively from electronic medical records using a standardized data collection sheet. Demographic, clinical, laboratory, disease activity, damage, and treatment data were collected. Disease activity was assessed at the first available visit during the study period using the Systemic Lupus Erythematosus Disease Activity Index 2000 (SLEDAI-2K), and cumulative damage was assessed at the last available follow-up using the Systemic Lupus International Collaborating Clinics/American College of Rheumatology Damage Index (SDI) [23,24]. Medication exposure was recorded as ever-use during the available follow-up period. The oral corticosteroid maintenance dose was expressed as the prednisolone-equivalent dose (mg/day) and categorized into dose ranges.

### 2.3. Definitions and Hematologic Phenotype Groups

Leukopenia was defined as a white blood cell count < 4.0 × 10^9^/L, and lymphopenia was defined as an absolute lymphocyte count < 1.0 × 10^9^/L, consistent with the 2012 Systemic Lupus International Collaborating Clinics classification thresholds [4]. Neutropenia was defined as an absolute neutrophil count < 1.5 × 10^9^/L and classified according to the lowest documented absolute neutrophil count during follow-up as mild (1.0 to <1.5 × 10^9^/L), moderate (0.5 to <1.0 × 10^9^/L), or severe (<0.5 × 10^9^/L), consistent with commonly used adult and SLE cohort definitions [8,25,26]. Ever-leukopenia, ever-neutropenia, and ever-lymphopenia were defined as at least one documented episode during follow-up. Cytopenia status was determined from all available complete blood count measurements recorded during the study follow-up period. Persistent neutropenia and persistent lymphopenia were defined as abnormalities documented at two or more separate visits at least 6 months apart. The 6-month interval was selected to distinguish persistent abnormalities from transient fluctuations and is consistent with previous SLE literature defining chronic neutropenia over at least 6 months [21]. The same interval was applied to lymphopenia for consistency.

Patients were classified into four hematologic phenotype groups based on their ever-neutropenia and ever-lymphopenia status: no neutropenia or lymphopenia, isolated neutropenia, isolated lymphopenia, and combined neutropenia and lymphopenia. In the ever-neutropenia regression model, patients without neutropenia were the reference group. In the ever-lymphopenia regression model, patients without lymphopenia were the reference group. For analyses of factors associated with ever-neutropenia, the outcome included isolated neutropenia or combined neutropenia and lymphopenia. For analyses of factors associated with ever-lymphopenia, the outcome included isolated lymphopenia or combined neutropenia and lymphopenia.

### 2.4. Statistical Analysis

Descriptive statistics are reported as means and standard deviations for normally distributed continuous variables, medians with ranges for non-normally distributed variables, and frequencies with percentages for categorical variables. Comparisons across the four neutropenia/lymphopenia phenotype groups were performed using one-way analysis of variance or the Kruskal–Wallis test for continuous variables, depending on distribution, and Pearson’s chi-square test or Fisher’s exact test for categorical variables, as appropriate.

Multivariable logistic regression analyses were performed to identify factors associated with ever-neutropenia and ever-lymphopenia. Variables were selected a priori based on clinical relevance and previously reported associations. Models included age at SLE diagnosis, sex, follow-up duration, SLEDAI-2K score, anti-Smith antibody status, renal involvement, and exposure to azathioprine (AZA), mycophenolate mofetil (MMF), rituximab, and belimumab. All variables were entered simultaneously. Descriptive analyses were based on available data, whereas multivariable regression analyses used complete-case analysis. Adjusted odds ratios (aORs) with 95% confidence intervals (CIs) were reported. All statistical tests were two-sided, and *p* < 0.05 was considered statistically significant. Data were analyzed using IBM SPSS Statistics (version 28.0; IBM Corp., Armonk, NY, USA).

### 2.5. Ethical Considerations

This study was approved by the Institutional Review Board of King Salman Bin Abdulaziz Medical City, Medina, Saudi Arabia, on 20 May 2025 (IRB log no. 25-040). Additionally, this study received expedited approval with a waiver of informed consent because of its retrospective design. The Institutional Review Board is registered with the National Committee of Bioethics in Saudi Arabia (H-03-M-11).

## 3. Results

### 3.1. Prevalence and Patterns of Leukopenia, Neutropenia, and Lymphopenia

Of the 353 patients included in the study, 141 (39.9%) experienced leukopenia at least once during follow-up (Table 1). Based on the cytopenia phenotype, 218 patients (61.8%) had neither neutropenia nor lymphopenia, 47 (13.3%) had isolated neutropenia, 47 (13.3%) had isolated lymphopenia, and 41 (11.6%) had both neutropenia and lymphopenia.

Ever-neutropenia was identified in 88 patients (24.9%). Persistent neutropenia, defined as neutropenia documented at two or more separate visits at least 6 months apart, occurred in 22 patients (6.2%). Based on the lowest recorded absolute neutrophil count, mild neutropenia was most common (51 patients; 58.0%), followed by moderate neutropenia (34 patients; 38.6%). Only three patients (3.4%) had severe neutropenia. Ever-lymphopenia occurred with the same overall frequency (88 patients; 24.9%), and persistent lymphopenia was identified in 23 patients (6.5%) (Table 1).

### 3.2. Characteristics by Cytopenia Phenotype

Selected demographic, clinical, laboratory, serologic, and treatment characteristics according to hematologic phenotype are presented in Table 2, with the complete comparison provided in Table S1. Mean age differed across groups and was lowest in patients with combined neutropenia and lymphopenia (36.2 years), followed by those with isolated lymphopenia (38.3 years), isolated neutropenia (40.5 years), and neither neutropenia nor lymphopenia (44.6 years; *p* = 0.002). Age at SLE diagnosis also differed across groups, ranging from 27.1 years in the combined neutropenia and lymphopenia group to 35.1 years in the group with neither abnormality (*p* = 0.002). Mean body mass index was highest in patients with neither neutropenia nor lymphopenia and lowest in those with both abnormalities (28.5 vs. 24.9 kg/m^2^; *p* < 0.001). Disease duration, follow-up duration, and number of hospital visits did not differ significantly across groups.

Median SLEDAI-2K scores were comparable across groups (*p* = 0.13). SDI scores differed significantly across groups (*p* = 0.021). The median number of American College of Rheumatology criteria fulfilled was highest in the combined neutropenia and lymphopenia group and lowest in the group with neither abnormality (5.0 vs. 3.0; *p* < 0.001).

Several laboratory parameters differed significantly across phenotype groups. Median hemoglobin was lowest in the isolated lymphopenia group (11.0 g/dL; *p* = 0.027). Autoimmune hemolytic anemia was most frequent in the isolated lymphopenia and combined neutropenia and lymphopenia groups (21.3% and 17.1%, respectively; *p* = 0.002). The mean platelet count was lower in groups with lymphopenia and was lowest in the combined neutropenia and lymphopenia group (235.7 × 10^3^/µL; *p* = 0.015). Anti-Smith positivity was highest in the combined neutropenia and lymphopenia group (45.0%), compared with 15.0% in patients with neither abnormality (*p* < 0.001). The frequency of low complement C3 did not differ significantly across groups (*p* = 0.076), and no significant differences were observed in anti-dsDNA or anti-SSA/SSB positivity.

Azathioprine use varied across groups and was most frequent in the combined neutropenia and lymphopenia group (46.3%), followed by the isolated lymphopenia group (44.7%; *p* = 0.027). MMF use was highest in the isolated lymphopenia group (57.4%; *p* < 0.001). Cyclophosphamide exposure also differed across groups (*p* = 0.012) and was highest in the isolated lymphopenia group (14.9%); none of the patients with combined neutropenia and lymphopenia had received cyclophosphamide. Rituximab use was more frequent in groups with lymphopenia, whether isolated or combined with neutropenia, than in patients with neither abnormality (approximately 17% in both lymphopenia groups vs. 3.7%; *p* < 0.001). Hydroxychloroquine use, prednisone dose, and belimumab use were similar across groups.

### 3.3. Factors Associated with Ever-Neutropenia and Ever-Lymphopenia

**Figure 1:**
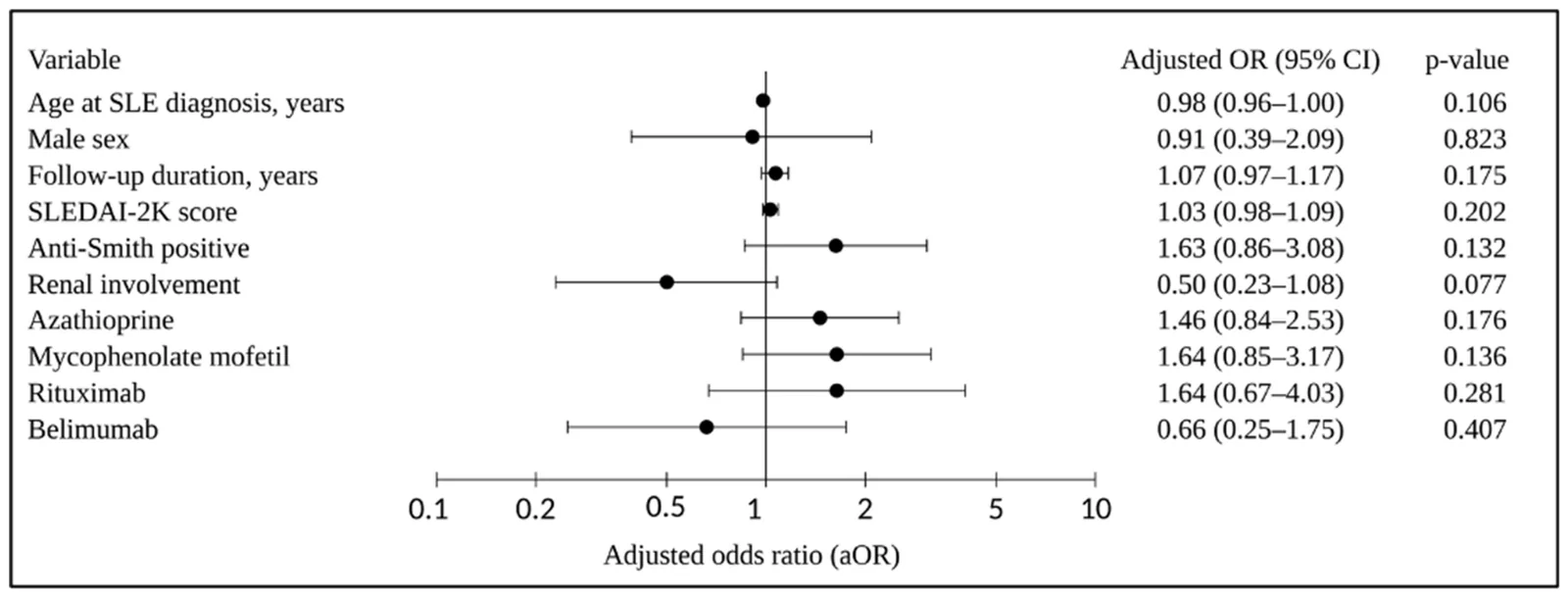
Forest plot of factors associated with ever-neutropenia in patients with systemic lupus erythematosus. The multivariable analysis included 327 patients; 84 had ever-neutropenia. Medication variables represent ever-use during follow-up. aOR, adjusted odds ratio; CI, confidence interval; SLEDAI-2K, Systemic Lupus Erythematosus Disease Activity Index 2000.

**Figure 2:**
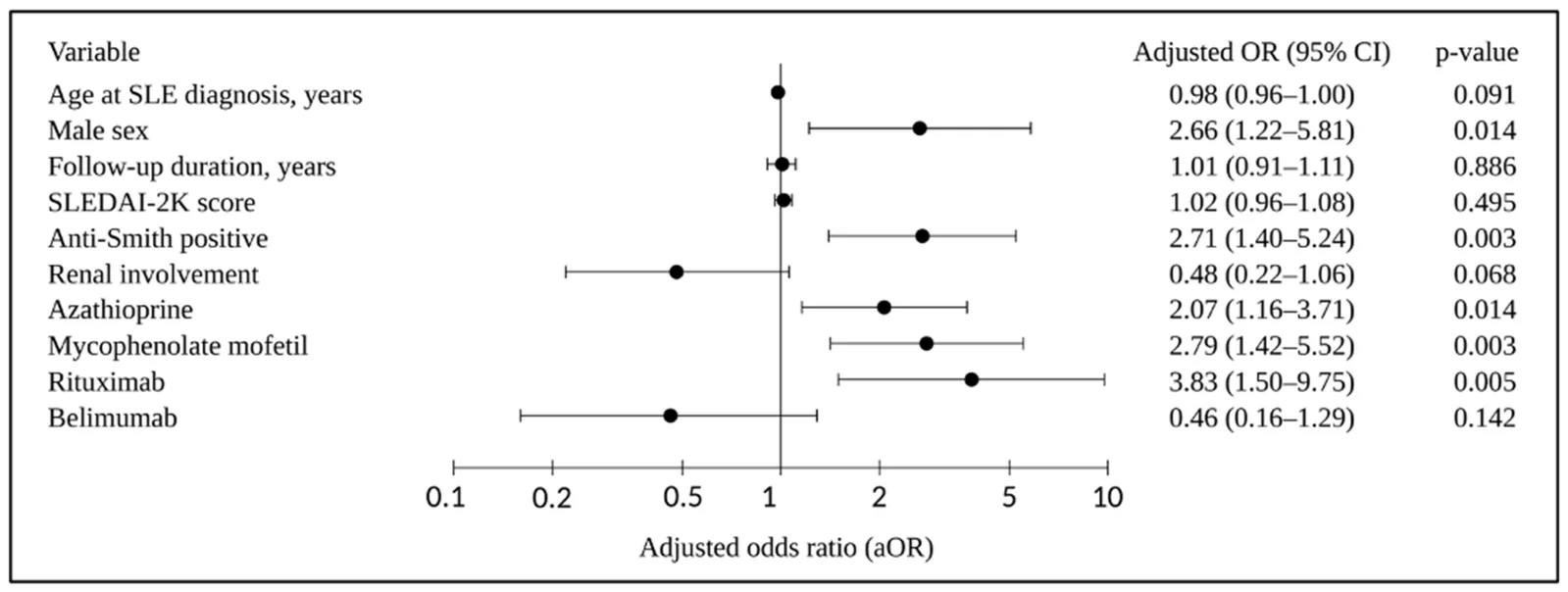
Forest plot of factors associated with ever-lymphopenia in patients with systemic lupus erythematosus. The multivariable analysis included 327 patients; 83 had ever-lymphopenia. Medication variables represent ever-use during follow-up. aOR, adjusted odds ratio; CI, confidence interval; SLEDAI-2K, Systemic Lupus Erythematosus Disease Activity Index 2000.

Multivariable logistic regression results are presented in Figure 1 and Figure 2. Complete-case regression analyses included 327 patients; 84 had ever-neutropenia, and 83 had ever-lymphopenia. The remaining 26 patients had missing data for age at SLE diagnosis (n = 12) and/or anti-Smith antibody status (n = 15), with one patient missing both. Excluded patients were older and had lower SLEDAI-2K scores; no significant differences were observed in the other variables included in the models or in the frequencies of ever-neutropenia and ever-lymphopenia.

In the multivariable model for ever-neutropenia, none of the examined variables was independently associated with ever-neutropenia (Figure 1). SLEDAI-2K score was not independently associated with either ever-neutropenia or ever-lymphopenia. Male sex (aOR 2.66, 95% CI 1.22–5.81; *p* = 0.014), anti-Smith antibody positivity (aOR 2.71, 95% CI 1.40–5.24; *p* = 0.003), AZA use (aOR 2.07, 95% CI 1.16–3.71; *p* = 0.014), MMF use (aOR 2.79, 95% CI 1.42–5.52; *p* = 0.003), and rituximab use (aOR 3.83, 95% CI 1.50–9.75; *p* = 0.005) were independently associated with ever-lymphopenia (Figure 2).

## 4. Discussion

We investigated the prevalence of neutropenia and lymphopenia and their associated factors in patients with SLE in a multicenter study conducted in Saudi Arabia. Leukopenia was observed at least once in 39.9% of patients, while ever-neutropenia and ever-lymphopenia were each observed in 24.9%. Persistent neutropenia and lymphopenia were uncommon.

The prevalence of neutropenia in our cohort was within the range reported in the systematic review by Carli et al. [13]. Previous cohort studies have reported variable frequencies, including 21% in a large European SLE cohort study, 12.5% in the Asia Pacific Lupus Collaboration cohort study, and 12% in a Swedish longitudinal cohort study [15,21,26]. Ethnic background may partly contribute to the variation in reported prevalence, as benign ethnic neutropenia is more common in Middle Eastern and African populations [27,28]. Because benign ethnic neutropenia was not specifically assessed in our cohort, its contribution to the frequency of mild neutropenia cannot be determined. This may be relevant when interpreting the frequency of mild neutropenia in this Saudi population. Most neutropenia in our study was mild; persistent neutropenia occurred in only 6.2% of patients, and severe neutropenia was rare. Similar findings have been reported in other SLE cohorts, in which chronic or severe neutropenia was observed in only a small number of patients [21,29,30]. Isolated neutropenia was observed in 13.3% of our patients, compared with 1.7% in the Toronto Lupus Cohort. This discrepancy may reflect methodological differences, as the Toronto study required isolated neutropenia on at least two consecutive visits, whereas our study used an ever-based definition [31].

Data on the prevalence of neutropenia in Saudi patients with SLE are limited, as most regional studies have reported leukopenia broadly without detailed information on neutropenia prevalence or severity [14]. In multivariable analysis, none of the examined variables, including SLEDAI-2K and immunosuppressive exposure, was independently associated with ever-neutropenia. In the Lupus BioBank of the Upper Rhine cohort, neutropenia was associated mainly with lymphopenia and thrombocytopenia, and chronic neutropenia was associated with anti-Ro/SSA positivity. SLEDAI and immunosuppressive treatment were not associated with neutropenia [21]. The Swedish longitudinal study also found that most neutropenic episodes occurred with other hematologic abnormalities, and anti-SSA antibodies were present in 62% of patients with neutropenia [26]. These findings differ from those of the Asia Pacific Lupus Collaboration cohort study, in which neutropenia was associated with serologic activity, elevated erythrocyte sedimentation rate, and use of rituximab and leflunomide [15]. In our study, SLEDAI-2K was assessed at the first available visit, and medication exposure was recorded as ever-use; therefore, their temporal relationship with neutropenia could not be determined.

Lymphopenia, which is recognized as the most common white blood cell abnormality in SLE, was observed at least once in 24.9% of patients in our cohort, a rate lower than reported in many previous studies [26,29,32]. A systematic review reported wide variation in the prevalence of lymphopenia among SLE cohorts, ranging from 15% to 82%, largely reflecting differences in definitions, timing of laboratory assessment, and study design [13]. Although lymphopenia has been linked to clinical and serologic disease activity in previous studies, the SLEDAI-2K score was not associated with ever-lymphopenia in our cohort [7,30,32,33]. This may be explained by comparable SLEDAI-2K scores at the first available visit across phenotype groups and the ever-based definition, as disease activity at that visit may not reflect activity at the time of lymphopenia. Therefore, an association between lymphopenia and disease activity when lymphopenia occurred cannot be excluded.

Ever-lymphopenia was independently associated with male sex, anti-Smith antibody positivity, and exposure to AZA, MMF, and rituximab. Male sex has also been linked to lymphopenia in previous SLE cohorts [34,35], although this finding should be interpreted cautiously because few men were included in our study. Anti-Smith positivity increased across phenotype groups from 15% in the no neutropenia or lymphopenia group to 45% in the combined neutropenia and lymphopenia group, and it remained independently associated with lymphopenia. This finding is consistent with findings from the PROFILE multiethnic SLE cohort, in which anti-Smith positivity was significantly associated with lymphopenia in multivariable analysis [36]. In the Asia Pacific Lupus Collaboration cohort, AZA and rituximab remained independently associated with lymphopenia, whereas MMF use did not remain significant after adjustment [15]. Gómez-Martín et al. identified a distinct T-cell profile in patients with AZA-associated lymphopenia, suggesting that treatment-associated lymphopenia may differ immunologically from lymphopenia related to active SLE [37]. The Korean Lupus Network registry found no significant difference in the frequency of lymphopenia between the MMF and non-MMF groups during follow-up [38]. Because treatment exposure was recorded as ever-use in our study, we could not determine whether AZA, MMF, or rituximab use preceded lymphopenia. These associations should therefore not be interpreted as causal treatment effects and may instead reflect greater immunosuppressive treatment in patients with more severe disease.

This study had several limitations. Its retrospective design limited assessment of the temporal relationships among cytopenias, disease activity, and treatment exposure. Medication exposure was recorded as ever-use, without information on cumulative dose, treatment sequence, or exposure at the time of cytopenia. Preanalytical variability, including delayed sample processing, could not be assessed, and transient factors may have influenced lymphocyte and neutrophil counts. Earlier episodes may have been missed because follow-up was shorter than disease duration in some patients, while variable complete blood count monitoring and ever-based definitions may have captured transient abnormalities. Lymphocyte subset analyses and functional immune-cell assessments were not available. Benign ethnic neutropenia was not assessed, and residual confounding by disease severity and treatment indication cannot be excluded. Center-related differences in patient characteristics, treatment practices, and laboratory monitoring were not evaluated.

Despite these limitations, to our knowledge, this is the first multicenter study from Saudi Arabia to report the prevalence and patterns of neutropenia and lymphopenia separately and examine their associated factors. It provides data from the Middle East, where detailed studies of these abnormalities remain limited. The multicenter design, standardized laboratory definitions, and classification into separate hematologic phenotypes allowed neutropenia and lymphopenia to be assessed individually rather than grouped under the broad term leukopenia. Multivariable analysis identified demographic, serologic, and treatment-related associations with ever-lymphopenia, whereas none of the examined variables was independently associated with ever-neutropenia.

## 5. Conclusions

Leukopenia was common, and ever-neutropenia and ever-lymphopenia each occurred in approximately one-quarter of patients with SLE. Persistent cytopenias were uncommon, and severe neutropenia was rare. Ever-neutropenia showed no independent associations, whereas ever-lymphopenia was associated with demographic, serologic, and treatment-related factors. These differences support the separate reporting and evaluation of neutropenia and lymphopenia in SLE and should be considered when interpreting hematologic abnormalities during clinical follow-up.

## Figures and Tables

**Table 1:** Prevalence and patterns of leukopenia, neutropenia, and lymphopenia in patients with systemic lupus erythematosus.

Characteristic	Total Cohort (n = 353)
Overall leukopenia	
Ever-leukopenia during follow-up, n (%)	141 (39.9%)
Neutropenia/lymphopenia phenotype, n (%)	
No neutropenia or lymphopenia	218 (61.8%)
Isolated neutropenia	47 (13.3%)
Isolated lymphopenia	47 (13.3%)
Combined neutropenia and lymphopenia	41 (11.6%)
Neutropenia	
Ever-neutropenia during follow-up, n (%)	88 (24.9%)
Persistent neutropenia (≥2 visits at least 6 months apart), n (%)	22 (6.2%)
Neutropenia severity *, n (%)	
Mild	51 (58%)
Moderate	34 (38.6%)
Severe	3 (3.4%)
Lymphopenia	
Ever-lymphopenia during follow-up, n (%)	88 (24.9%)
Persistent lymphopenia (≥2 visits at least 6 months apart), n (%)	23 (6.5%)

* Neutropenia severity percentages were calculated among the 88 patients with ever-neutropenia. Severity was classified according to the lowest absolute neutrophil count recorded during follow-up as mild (1.0 to <1.5 × 10^9^/L), moderate (0.5 to <1.0 × 10^9^/L), or severe (<0.5 × 10^9^/L).

**Table 2:** Selected characteristics of patients with systemic lupus erythematosus according to neutropenia/lymphopenia phenotype.

Characteristic	No Neutropenia or Lymphopenia (n = 218)	Isolated Neutropenia (n = 47)	Isolated Lymphopenia (n = 47)	Combined Neutropenia and Lymphopenia (n = 41)	*p*-Value
Age, years, mean (SD)	44.6 (17.0)	40.5 (15.7)	38.3 (15.0)	36.2 (12.6)	0.002
Female sex, n (%)	194 (89.0)	43 (91.5)	36 (76.6)	36 (87.8)	0.10
BMI, kg/m^2^, mean (SD)	28.5 (6.1)	26.4 (6.1)	25.3 (7.7)	24.9 (4.3)	<0.001
Age at SLE diagnosis, years, mean (SD)	35.1 (14.3)	32.4 (13.9)	30.3 (13.9)	27.1 (10.3)	0.002
Disease duration, years, mean (SD)	8.9 (7.5)	7.1 (4.9)	8.3 (8.3)	9.1 (7.4)	0.69
Follow-up duration, years, mean (SD)	3.0 (3.0)	3.5 (2.6)	3.2 (2.8)	3.1 (3.1)	0.45
SLEDAI-2K, median (min–max)	6.0 (0–52)	6.0 (0–20)	6.0 (0–16)	6.0 (0–22)	0.13
SLICC/ACR Damage Index (SDI), median (min–max)	0.0 (0–5)	0.0 (0–3)	0.0 (0–4)	0.0 (0–2)	0.021
ACR criteria fulfilled, median (min–max)	3.0 (0–8)	4.0 (1–7)	4.0 (2–8)	5.0 (2–9)	<0.001
Renal involvement, n (%)	49 (22.5)	10 (21.3)	15 (31.9)	7 (17.1)	0.39
Hemoglobin, g/dL, median (min–max)	12.0 (5–18.7)	12.0 (2.7–14.7)	11.0 (5.3–13.8)	11.9 (4.9–12.7)	0.027
Autoimmune hemolytic anemia, n (%)	12 (5.5)	4 (8.5)	10 (21.3)	7 (17.1)	0.002
Platelet count, ×10^3^/µL, mean (SD)	275.0 (87.2)	274.9 (73.3)	243.5 (91.4)	235.7 (62.0)	0.015
Low complement C3, n (%)	66 (31.7)	14 (29.8)	24 (53.3)	18 (46.2)	0.076
Anti-Smith positive, n (%)	31 (15.0)	5 (10.6)	9 (20.0)	18 (45.0)	<0.001
Azathioprine, n (%)	60 (27.5)	16 (34.0)	21 (44.7)	19 (46.3)	0.027
Mycophenolate mofetil, n (%)	57 (26.1)	18 (38.3)	27 (57.4)	14 (34.1)	<0.001
Cyclophosphamide, n (%)	9 (4.1)	3 (6.4)	7 (14.9)	0 (0.0)	0.012
Rituximab, n (%)	8 (3.7)	3 (6.4)	8 (17.0)	7 (17.1)	<0.001
Belimumab, n (%)	19 (8.7)	3 (6.4)	3 (6.4)	4 (9.8)	0.92

Continuous variables were compared using one-way analysis of variance or the Kruskal–Wallis test, as appropriate, and categorical variables using Pearson’s chi-square test or Fisher’s exact test. ACR, American College of Rheumatology; BMI, body mass index; SD, standard deviation; SDI, Systemic Lupus International Collaborating Clinics/American College of Rheumatology Damage Index; SLEDAI-2K, Systemic Lupus Erythematosus Disease Activity Index 2000.

## Data Availability

The data that support the findings of this study are available from the corresponding author upon reasonable request.

## Supplementary Materials

The following supporting information can be downloaded at https://www.mdpi.com/article/10.3390/jcm15166481/s1, Table S1: Clinical characteristics of patients with systemic lupus erythematosus according to neutropenia/lymphopenia phenotype.

## Abbreviations

The following abbreviations are used in this manuscript:

ACR	American College of Rheumatology
ANC	Absolute neutrophil count
aOR	Adjusted odds ratio
AZA	Azathioprine
BMI	Body mass index
MMF	Mycophenolate mofetil
SDI	Systemic Lupus International Collaborating Clinics/American College of Rheumatology Damage Index
SLE	Systemic lupus erythematosus
SLEDAI-2K	Systemic Lupus Erythematosus Disease Activity Index 2000
SLICC	Systemic Lupus International Collaborating Clinics
