# Supplementary material for: Neutropenia and Lymphopenia in Systemic Lupus Erythematosus: Distinct Phenotypes and Associated Factors in a Saudi Multicenter Study
Source: J Clin Med. 2026 Aug 21;15(16):6481. doi: 10.3390/jcm15166481 (PMC13513896; doi:10.3390/jcm15166481)
Supplement: Supplementary file 1 [file jcm-15-06481-s001.zip › jcm-4481959-supplementary.pdf]

Supplementary materials

Supplementary Table S1. Clinical characteristics of patients with systemic lupus erythematosus according to neutropenia/lymphopenia phenotype

Characteristic	No neutropenia or lymphopenia (n = 218)	Isolated neutropenia (n = 47)	Isolated lymphopenia (n = 47)	Combined neutropenia and lymphopenia (n = 41)	p-value
**Demographics**					
Age, years, mean (SD)	44.6 (17.0)	40.5 (15.7)	38.3 (15.0)	36.2 (12.6)	0.002
Female sex, n (%)	194 (89.0)	43 (91.5)	36 (76.6)	36 (87.8)	0.10
Saudi nationality, n (%)	201 (92.2)	46 (97.9)	45 (95.7)	41 (100.0)	0.16
BMI, kg/m², mean (SD)	28.5 (6.1)	26.4 (6.1)	25.3 (7.7)	24.9 (4.3)	<0.001
Age at SLE diagnosis, years, mean (SD)	35.1 (14.3)	32.4 (13.9)	30.3 (13.9)	27.1 (10.3)	0.002
Disease duration, years, mean (SD)	8.9 (7.5)	7.1 (4.9)	8.3 (8.3)	9.1 (7.4)	0.69
Follow-up duration, years, mean (SD)	3.0 (3.0)	3.5 (2.6)	3.2 (2.8)	3.1 (3.1)	0.45
Hospital visits >10, n (%)	89 (40.8)	26 (55.3)	20 (42.6)	17 (41.5)	0.30
**Disease Activity and Damage**					
SLEDAI-2K, median (min–max)	6.0 (0–52)	6.0 (0–20)	6.0 (0–16)	6.0 (0–22)	0.13
SLICC/ACR Damage Index (SDI), median (min–max)	0.0 (0–5)	0.0 (0–3)	0.0 (0–4)	0.0 (0–2)	0.021
ACR criteria fulfilled, median (min–max)	3.0 (0–8)	4.0 (1–7)	4.0 (2–8)	5.0 (2–9)	<0.001
**ACR Classification Criteria, n (%)**					
Malar rash	62 (28.4)	20 (42.6)	10 (21.3)	15 (36.6)	0.10
Discoid rash	10 (4.6)	2 (4.3)	4 (8.5)	4 (9.8)	0.40
Photosensitivity	52 (23.9)	11 (23.4)	10 (21.3)	7 (17.1)	0.81
Oral ulcers	45 (20.6)	5 (10.6)	12 (25.5)	13 (31.7)	0.093
Arthritis	100 (45.9)	24 (51.1)	18 (38.3)	22 (53.7)	0.47
Serositis	8 (3.7)	3 (6.4)	3 (6.4)	5 (12.2)	0.12
Renal involvement	49 (22.5)	10 (21.3)	15 (31.9)	7 (17.1)	0.39
Neurologic disorder	4 (1.8)	1 (2.1)	2 (4.3)	2 (4.9)	0.38
Hematologic disorder	67 (30.7)	42 (89.4)	42 (89.4)	39 (95.1)	<0.001
Immunologic disorder	153 (70.2)	38 (80.9)	40 (85.1)	33 (80.5)	0.081
ANA positive	183 (83.9)	41 (87.2)	38 (80.9)	37 (90.2)	0.61
**Comorbidities, n (%)**					
Diabetes mellitus	33 (15.5)	4 (8.5)	6 (13.0)	0 (0.0)	0.039
Chronic kidney disease	16 (7.3)	4 (8.5)	7 (14.9)	2 (4.9)	0.33
**Laboratory Values**					
Hemoglobin, g/dL, median (min–max)	12.0 (5–18.7)	12.0 (2.7–14.7)	11.0 (5.3–13.8)	11.9 (4.9–12.7)	0.027
Autoimmune hemolytic anemia, n (%)	12 (5.5)	4 (8.5)	10 (21.3)	7 (17.1)	0.002
Platelet count, ×10³/µL, mean (SD)	275.0 (87.2)	274.9 (73.3)	243.5 (91.4)	235.7 (62.0)	0.015
**Serological Profile, n (%)**					
Low complement C3	66 (31.7)	14 (29.8)	24 (53.3)	18 (46.2)	0.076
Low complement C4	45 (21.8)	15 (31.9)	17 (38.6)	14 (36.8)	0.13
Anti-dsDNA positive	132 (75.0)	34 (79.1)	35 (83.3)	30 (83.3)	0.53
Anti-Smith positive	31 (15.0)	5 (10.6)	9 (20.0)	18 (45.0)	<0.001
Anti-SSA positive	51 (24.8)	15 (31.9)	13 (28.9)	11 (27.5)	0.76
Anti-SSB positive	15 (7.3)	6 (12.8)	1 (2.2)	5 (12.5)	0.17
**Treatment Exposures, n (%)**					
Prednisone dose, mg, mean (SD)	6.4 (10.6)	5.5 (8.8)	7.1 (11.3)	4.4 (3.7)	0.76
Prednisone ever used	150 (68.8)	31 (66.0)	37 (78.7)	32 (80.0)	0.26
≤5 mg/day	98 (48.8)	20 (47.6)	26 (61.9)	25 (67.6)	0.37
>5–10 mg/day	15 (7.5)	1 (2.4)	2 (4.8)	3 (8.1)	0.37
>10 mg/day	20 (10.0)	5 (11.9)	4 (9.5)	1 (2.7)	0.37
Hydroxychloroquine	164 (75.2)	33 (70.2)	36 (76.6)	31 (75.6)	0.89
Azathioprine	60 (27.5)	16 (34.0)	21 (44.7)	19 (46.3)	0.027
Mycophenolate mofetil	57 (26.1)	18 (38.3)	27 (57.4)	14 (34.1)	<0.001
Cyclophosphamide	9 (4.1)	3 (6.4)	7 (14.9)	0 (0.0)	0.012

Any biologic therapy	25 (11.5)	6 (12.8)	8 (17.0)	10 (24.4)	0.15
Rituximab	8 (3.7)	3 (6.4)	8 (17.0)	7 (17.1)	<0.001
Belimumab	19 (8.7)	3 (6.4)	3 (6.4)	4 (9.8)	0.92

Values are mean (SD), median (min–max), or n (%), as indicated. p-values were obtained using one-way analysis of variance or the Kruskal–Wallis test for continuous variables and Pearson's chi-square test or Fisher's exact test for categorical variables, as appropriate. ACR, American College of Rheumatology; ANA, antinuclear antibody; BMI, body mass index; SD, standard deviation; SDI, Systemic Lupus International Collaborating Clinics/American College of Rheumatology Damage Index; SLE, systemic lupus erythematosus; SLEDAI-2K, Systemic Lupus Erythematosus Disease Activity Index 2000.
